# Biomarkers Involved in the Pathogenesis of Hemophilic Arthropathy

**DOI:** 10.3390/ijms25189897

**Published:** 2024-09-13

**Authors:** Oana Viola Badulescu, Dragos-Viorel Scripcariu, Minerva Codruta Badescu, Manuela Ciocoiu, Maria Cristina Vladeanu, Carmen Elena Plesoianu, Andrei Bojan, Dan Iliescu-Halitchi, Razvan Tudor, Bogdan Huzum, Otilia Elena Frasinariu, Iris Bararu-Bojan

**Affiliations:** 1 Department of Pathophysiology, University of Medicine and Pharmacy Grigore T. Popa, 700115 Iasi, Romania; oana.badulescu@umfiasi.ro (O.V.B.); manuela.ciocoiu@umfiasi.ro (M.C.); maria.apavaloaie@umfiasi.ro (M.C.V.); iris.bararu@umfiasi.ro (I.B.-B.); 2 Department of Surgical Sciences, University of Medicine and Pharmacy Grigore T. Popa, 700115 Iasi, Romania; rc_tudor@yahoo.com (R.T.); bogdan.huzum@umfiasi.ro (B.H.); 3 Department of Internal Medicine, University of Medicine and Pharmacy Grigore T. Popa, 700115 Iasi, Romania; minerva.badescu@umfiasi.ro (M.C.B.); carmen-elena.plesoianu@umfiasi.ro (C.E.P.); halitchi.iliescud@umfiasi.ro (D.I.-H.); 4 Department of Pediatry, University of Medicine and Pharmacy Grigore T. Popa, 700115 Iasi, Romania; frasinariu.otilia@umfiasi.ro

**Keywords:** hemophilic arthropathy, biomarkers in hemophilic arthropathy, joint lesions in hemophilia

## Abstract

Hemophilia, which is a rare disease, results from congenital deficiencies of coagulation factors VIII and IX, respectively, leading to spontaneous bleeding into joints, resulting in hemophilic arthropathy (HA). HA involves complex processes, including synovial proliferation, angiogenesis, and tissue remodeling. Despite ongoing research, factors contributing to HA progression, especially in adults with severe HA experiencing joint pain, remain unclear. Blood markers, particularly collagen-related ones, have been explored to assess joint health in hemophilia. For example, markers like CTX-I and CTX-II reflect bone and cartilage turnover, respectively. Studies indicate elevated levels of certain markers post-bleeding episodes, suggesting joint health changes. However, longitudinal studies on collagen turnover and basement membrane or endothelial cell markers in relation to joint outcomes, particularly during painful episodes, are scarce. Given the role of the CX3CL1/CX3XR1 axis in arthritis, other studies investigate its involvement in HA. The importance of different inflammatory and bone damage biomarkers should be assessed, alongside articular cartilage and synovial membrane morphology, aiming to enhance understanding of hemophilic arthropathy progression.

## 1. Introduction

Hemophilia is an X-linked, inherited coagulation disorder characterized by deficiencies in specific plasma glycoproteins that are crucial for blood clotting. The disorder manifests in two primary forms: hemophilia A, caused by reduced levels of coagulation factor VIII (FVIII), and hemophilia B (also known as Christmas disease), caused by reduced levels of coagulation factor IX (FIX) [1]. These deficiencies impede the blood clotting process, leading to a propensity for prolonged bleeding.

One of the most common and severe complications associated with hemophilia is hemarthrosis, which refers to bleeding into the joints. This condition is particularly prevalent in individuals with hemophilia [2]. It is estimated that hemarthrosis accounts for 70–80% of all bleeding episodes in patients with hemophilia [3]. Repeated joint bleeds can lead to chronic pain, inflammation, and long-term joint damage, significantly impacting the quality of life for those affected. Managing hemarthrosis often requires a combination of prophylactic treatment with clotting factor concentrates, physical therapy, and sometimes surgical intervention to mitigate joint damage and maintain mobility [4].

The syndrome of joint manifestations that occurs in the course of hemophilia is known as hemophilic arthropathy (HA). Research indicates that around 90% of patients with severe hemophilia A (defined as having FVIII levels below 0.01 IU/mL in serum) experience joint bleeding by the age of 4.4 years. This early onset of joint bleeding significantly contributes to the development of HA, which is characterized by chronic joint pain, swelling, and reduced mobility.

Despite the availability of effective prophylactic factor replacement therapy (FRT) in many parts of the world, including highly developed countries [3], advanced and fully developed HA remains a significant global health issue. Prophylactic FRT involves the regular infusion of clotting factor concentrates to prevent bleeding episodes and protect joint health. However, the progression of HA can still occur, especially in severe cases of hemophilia, due to the development of inhibitors. These inhibitors are antibodies that neutralize the infused clotting factors (anti-FVIII/FIX), rendering the treatment less effective or even ineffective.

The management of HA requires a multifaceted approach. In addition to FRT, patients may benefit from physical therapy to maintain joint function and mobility. In cases where inhibitors are present, immune tolerance induction (ITI) therapy may be used to reduce inhibitor levels. Advanced cases of HA might also necessitate orthopedic interventions, such as synovectomy or joint replacement surgery, to alleviate pain and improve joint function [5].

Globally, efforts are ongoing to improve access to comprehensive hemophilia care, including prophylactic FRT and multidisciplinary management strategies, to reduce the incidence and severity of HA and enhance the quality of life for patients with hemophilia.

Our current understanding of the factors contributing to the progression of HA remains incomplete. It is particularly noteworthy that adults with hemophilia, especially those with advanced HA, frequently experience episodes of joint pain. These episodes can occur with or without accompanying joint bleeding, adding complexity to the condition. The precise impact of these pain episodes on the molecular mechanisms driving the progression of HA is not yet fully understood.

Research is ongoing to elucidate the underlying mechanisms that cause joint damage in hemophilia. The interaction between recurrent bleeding and subsequent inflammation is believed to play a crucial role in the deterioration of joint health. Inflammation resulting from bleeding episodes leads to synovial hypertrophy, cartilage degradation, and bone erosion, all of which contribute to the development and progression of HA.

Moreover, the role of subclinical bleeds—minor bleeding episodes that do not cause noticeable symptoms—remains a significant area of investigation. These bleeds can contribute to joint damage over time, even in the absence of acute pain or swelling. Understanding how these subclinical events influence the molecular pathways involved in HA is critical for developing more effective treatment strategies [6].

There is also interest in the potential genetic and environmental factors that may influence the severity and progression of HA. Differences in genetic makeup may affect an individual’s susceptibility to joint damage and response to treatment. Environmental factors, such as physical activity levels and access to comprehensive hemophilia care, also play a role in the disease’s trajectory.

To address these gaps in knowledge, researchers are employing advanced imaging techniques, molecular biology, and clinical studies to gain a deeper understanding of HA. The goal is to identify biomarkers that can predict disease progression and to develop targeted therapies that can prevent or mitigate joint damage more effectively. As our understanding of HA improves, it holds the promise of better management and improved outcomes for individuals living with hemophilia [7].

Regular prophylactic intravenous infusion of factor VIII is the standard treatment for individuals with severe hemophilia A. However, due to the relatively short half-life of factor VIII, more than two infusions per week are required to maintain protective trough levels. This regimen places a significant treatment burden on patients and can result in inadequate care for those who struggle to adhere to it. Even with regular prophylaxis, both clinical and subclinical bleeding events may still occur. Therefore, treatments with greater efficacy and reduced burden are needed.

Emicizumab (Hemlibra, F. Hoffmann–La Roche) is a recombinant, humanized, bispecific monoclonal antibody that bridges activated factor IX and factor X to compensate for the missing activated factor VIII, thereby restoring hemostasis. Its efficacy has been demonstrated in individuals with hemophilia A who have developed neutralizing anti-factor VIII alloantibodies (inhibitors), and it is administered once a week. Therefore, administering emicizumab once weekly or every two weeks resulted in significantly lower bleeding rates compared to no prophylaxis. In an intraindividual comparison, once-weekly emicizumab prophylaxis was associated with a significantly reduced bleeding rate compared to previous factor VIII prophylaxis [8].

## 2. Literature Search

We conducted systematic research in order to identify the key biomarkers associated with hemophilia arthropathy and to elucidate the pathogenesis of this condition.

To achieve this, we performed a systematic search on PubMed to gather relevant studies concerning biomarkers in hemophilic arthropathy. We used specific keywords such as “hemophilic arthropathy”, “hemophilia”, “biomarkers in hemophilia”, and “biomarkers in hemophilic arthropathy”.

Our search strategy was designed to include a broad range of studies to ensure comprehensive coverage of the topic. We set the following inclusion criteria: studies must have been published in English to ensure accurate interpretation and analysis; studies must have involved human subjects to ensure clinical relevance. Single-case reports were excluded to focus on studies with broader applicability and robust data.

Through this methodical approach, we aimed to compile a comprehensive and accurate overview of the current understanding of hemophilic arthropathy and its biomarkers. Our review not only highlights the most critical biomarkers but also discusses their potential roles in the pathogenesis and progression of the disease. Additionally, we explore how these biomarkers can be used in clinical practice to improve the diagnosis, monitoring, and treatment of hemophilic arthropathy.

By synthesizing findings from multiple studies, our review provides valuable insights into the molecular mechanisms underlying hemophilic arthropathy and offers a foundation for future research aimed at developing targeted therapies and improving patient outcomes.

## 3. Pathophysiology

The pathophysiology of blood-induced joint disease, such as HA, remains not fully understood. Some evidence suggests that the pathobiology of HA may be similar to that of rheumatoid arthritis (RA), as both conditions feature chronic proliferative synovitis and cartilage destruction. Additionally, the presence of metalloprotease enzymes, inflammatory cells, and inflammatory cytokines (such as interleukin-1 (IL-1) and tumor necrosis factor alpha (TNF-α)) has been observed in the synovium of both HA and RA.

However, a key difference between these diseases lies in the origin of inflammation: RA is driven by autoimmunity, while HA is triggered by blood-induced injury. In HA, synovial iron deposition resulting from blood in the joint is considered a primary factor in initiating and sustaining the inflammatory response and cell proliferation within the synovial membrane. Synovial macrophages responding to blood injury promote the inflammatory pathway of hemarthrosis and HA.

Despite these similarities, these factors alone do not fully explain the pathophysiology of HA. For instance, in hemochromatosis—a different arthropathy characterized by synovial iron deposition—significant synovial hyperplasia is not observed, although inflammatory cells and joint destruction can occur.

The distinguishing factor in HA is the presence of blood in the joint, directly contacting the synovial membrane and particularly affecting fibroblast-like synoviocytes (FLS), the unique resident cells in joints. This direct interaction between blood and the synovial membrane is pivotal in HA’s pathology, setting it apart from other forms of arthritis.

The presence of extravasated blood directly within the joint triggers an inflammatory response, initiating a sequence of immunopathological processes [6]. Within this blood are numerous active morphotic elements, including granulocytes, monocytes, and lymphocytes, which actively secrete inflammatory mediators [8].

Over time, macrophages and cells of the synovial membrane gradually absorb the blood and its associated metabolites. However, with recurrent episodes of hemarthrosis, the efficiency of immune cells and the synovial membrane in removing these metabolites diminishes. Consequently, toxic blood components such as iron (Fe^2+^) and hemosiderin accumulate within the synovial membrane cells.

This accumulation sets off a chain of pathological events, including degeneration of the articular cartilage and subchondral bone, proliferation of fibroblasts, and the formation of new blood vessels (angiogenesis). These processes collectively lead to hypertrophy of the synovial membrane [8,9,10].

The combined data on the pathophysiology of hemarthrosis (HA) and related arthritis suggest a clear link between the degeneration of joint tissues in the musculoskeletal system and a persistent, chronic inflammatory state [6]. This interconnectedness underscores the complex nature of joint diseases where ongoing inflammation plays a central role in tissue damage and dysfunction [11] (Table 1).

## 4. Chemokines and Cytokines

While certain cytokines have been implicated in the development of synovitis in hemophilic arthropathy, drawing parallels with rheumatoid arthritis (RA), studies demonstrating their precise functional role in the disease’s pathogenic cascade remain limited [12]. Initially described as a degenerative rather than inflammatory joint disorder, recent research suggests that hemophilic arthropathy shares similarities with both the degenerative joint damage seen in osteoarthritis (OA) and the chronic inflammatory processes of RA [13].

The extensive array of mediators implicated in HA pathogenesis includes cytokines, chemokines, and their associated receptors and signaling pathways. In a study conducted by Wojdasiewicz et al. on 40 patients, it was proven that the CX3CL1/CX3CR1 signaling axis plays a role in the development of degenerative joint lesions in patients with HA. They found a statistically significant increase in the average concentration of CX3CL1 in the serum of patients with HA compared to patients with osteoarthritis (OA). However, no statistically significant difference in CX3CL1 concentration was observed in other biofluids, such as synovial fluid, between patients with HA and patients with OA, despite a higher mean level in patients with HA. Immunohistochemical and histological analyses revealed CX3CR1 overexpression in the synovial membrane of patients with HA and OA, with no significant differences noted in articular cartilage slices between groups. However, there was an observable tendency towards increased extracellular matrix staining, although without significant intergroup differences. These findings suggested that inflammatory and degenerative processes mediated by CX3CR1 activation exhibit similar intensity in the synovial membrane of affected joints in both patient groups. Nonetheless, patients with HA demonstrated more pronounced activity of synovial membrane cells in CX3CL1 production and a systemic tendency towards higher serum concentrations compared to patients with OA. The similar level of CX3CR1 expression in synovial membrane slices between patients with HA and patients with OA may have reflected comparable clinical stages of joint disease development and the implementation of effective management strategies, including regular FRT administration and appropriate care programs aimed at minimizing hemarthrosis and joint-related exacerbations [14]. 

The “inflammasome”, a critical regulator of pro-inflammatory interleukin (IL)-1β maturation and secretion, has garnered attention in joint pathology [15]. Iron also plays a pivotal role by inducing the expression of several pro-inflammatory cytokines, including IL-1α, IL-6, and tumor necrosis factor (TNF)-α. Additionally, iron appears to contribute to the initiation of synovial pannus growth by disrupting the expression of key genes such as c-myc and mdm2, which govern synoviocyte proliferation. Synovitis, characterized by synovial tissue inflammation, involves hypertrophy, inflammatory cell migration, and substantial neo-angiogenesis. IL-1α, IL-6, IL-1β, and TNF-α activate monocytes/macrophages, initiating a catabolic response that includes the production of nitric oxide (NO), matrix metalloproteinases (MMPs), tissue plasminogen activator, and other matrix components [16,17,18]. These factors, in turn, influence T cells, fibroblasts, and osteoclasts through various inflammatory mediators, ultimately leading to degradation of articular cartilage and subchondral bone.

Mignot et al. proved that the cytokine expression profile analyzed using ELISPOT between HA-FLS and non-HA-FLS, both activated or not by LPS, appears to align with the inflammatory theory. Specifically, cytokines associated with innate immunity showed notable differences in expression between non-HA and HA-FLS. Additionally, HA-FLS were observed to be involved in processes related to bone remodeling and iron recycling, underscoring the pivotal role of FLS in hemophilic arthropathy pathophysiology. Interestingly, they observed that HA-FLS and RA-FLS did not secrete IL-1α following LPS treatment, whereas IL-1α was detected in supernatants from non-HA-FLS, THP-1 cell lines, and HR-FLS. The reasons for this paradoxical down-regulation of IL-1α in LPS-induced HA-FLS and RA-FLS remain unknown, despite the established role of IL-1β in the pathogenesis of HA [11,19].

TNF-α plays a significant role in inflammation in hemophilic arthropathy (HA), as highlighted in recent research focusing on the iRhom2/ADAM17/TNF-α pathway. It is also recognized as a crucial mediator of proliferative synovitis in hemophilia A.

In contrast to TNF-α and IL-1α, Mignot et al. observed elevated levels of IL-6 expression after LPS treatment in HA-FLS, consistent with previous findings suggesting the therapeutic potential of targeting IL-6 in HA. Interestingly, the cytokine profile of LPS-induced HA-FLS closely correlated with the potential post-transcriptional control of 30 selected miRNAs. Following LPS activation, HA-FLS up-regulated cytokines associated with macrophage polarization (also influenced by miR-1246) and modulated the TH17 pathway (miR-10b-5p), which is implicated in conditions like ankylosing spondylitis. Additionally, many of the other miRNAs may directly or indirectly affect inflammatory cytokine expression through pathways such as NFκB, PI3K, or JAK/STAT (notably miR-146 and miR-196b-5p, which exhibited the highest expression ratios) [20].

IL-1 triggers the activation of nuclear factor kappa-light-chain-enhancer of activated B cells (NF-κB) transcription factor, along with other factors like c-Jun N-terminal kinase (JNK) and p38 mitogen-activated protein kinases (p38MAPK). This cascade results in increased expression of various genes responsible for synthesizing enzymes, adhesion molecules, and inflammatory mediators such as cytokines and chemokines [4]. This mechanism parallels the role of NFκB in the development of synovitis and cartilage degeneration observed in osteoarthritis (OA) and rheumatoid arthritis (RA) [6]. Consistently underscoring the involvement of IL-1β in hemophilic arthropathy (HA) pathophysiology, several studies have documented significantly elevated IL-1β levels in histological sections of synovial membranes obtained during synovectomy or joint replacement from patients with HA compared to non-hemophilic individuals [3,4,11].

Furthermore, IL-1β can enhance transferrin-bound iron uptake by type B synoviocytes, leading to hemosiderin deposition and autocrine IL-1β secretion, thereby contributing to the development of chronic synovitis [21]. 

TNFα, a member of the tumor necrosis factor superfamily, plays a critical role in the pathophysiology of hemophilic arthropathy (HA). It induces catabolic processes in synovial joints and directly regulates intra-articular levels of FVIIIa, thereby modulating thrombomodulin (TM) expression [22]. Specifically, TNFα inhibits the synthesis of proteoglycans and collagen type II (COL2) by chondrocytes. It also promotes the expression of metalloproteases (MMP-1, MMP-3, MMP-13, and ADAMTS4), which play pivotal roles in joint catabolism [4,22]. Moreover, TNFα increases the risk of recurrent bleeding. It substantially reduces TM synthesis by synoviocytes, leading to elevated TM levels in synovial fluid due to neutrophil and cytokine activity on synovial cells. Recent studies indicate higher TM levels in patients with HA (56 ± 25 ng/mL) compared to healthy controls (39 ± 21 ng/mL). Normally, TM binds thrombin in a 1:1 ratio, activating protein C (PC), which inhibits coagulation by degrading factors FVa and FVIIIa [23,24]. This interplay between inflammatory mediators and hemostasis components helps explain persistent hemorrhagic processes despite FVIII replacement therapies.

Thus, IL-1β and TNFα, by triggering and exacerbating inflammatory damage and its consequences on joints, are pivotal in the pathophysiology of HA. Additionally, recent findings indicate elevated expression of the TNFα/TNF receptor (TNF-R) system in synovial tissue. Activation of this system may serve as a critical mediator of synovial proliferation, presenting a potential novel target for therapeutic intervention [25].

Moreover, a recent study demonstrated that similar to osteoarthritis (OA) and rheumatoid arthritis (RA), patients with hemophilic arthropathy (HA) exhibit increased levels of progranulin (PGRN), a molecule known for its protective role against the catabolic effects of TNFα [26]. This finding suggests potential future investigations into its role as a biomarker for monitoring disease activity (Table 2).

These cytokines and chemokines play diverse roles in the pathogenesis of HA, reflecting overlapping and unique mechanisms compared to RA and OA. Their dysregulation contributes to joint inflammation, cartilage degradation, and bone resorption, highlighting potential targets for therapeutic interventions specific to HA.

## 5. Soluble Adhesion Molecules

Several studies have shown that circulating concentrations of soluble adhesion molecules (CAMs), sE-selectin, and sP-selectin are elevated in conditions such as coronary artery disease, myocardial infarction, atherosclerosis, or hypertension [27,28].

### 5.1. sVCAM-1 Levels

Among patients with hemophilia A, the sVCAM-1 levels did not differ significantly between those with and without hypertension. This finding does not necessarily indicate that sVCAM-1 levels are independent of hypertension, as the study only included three patients with both hemophilia A and hypertension. Additionally, the influence of hypertension on sVCAM-1 levels may be overshadowed by factors related to hemophilia A. Previous studies have demonstrated that sVCAM-1 levels are significantly elevated in patients with hemodialysis, indicating chronic inflammation. Chronic hepatitis C virus (HCV) infection, which is common among patients with hemodialysis, can also trigger chronic inflammation. Levels of sICAM-1, sVCAM-1, and sE-selectin are higher in patients with hemodialysis who are anti-HCV-positive [29,30,31]. HCV infection is also highly prevalent in patients with hemophilia A. However, in this study, the sVCAM-1 levels among these patients did not show significant differences when compared to healthy volunteers and asymptomatic chronic hepatitis C carriers with minimal inflammatory activity. Notably, sVCAM-1 levels only increased significantly during the advanced fibrosis stage of HCV infection. This indicates that while HCV infection is common in hemophilia A patients, its impact on sVCAM-1 levels is not pronounced until the infection progresses to a more severe stage characterized by extensive liver fibrosis. Thus, monitoring sVCAM-1 levels could potentially serve as an indicator of disease progression in HCV-infected individuals, particularly in those with underlying conditions such as hemophilia A [32].

### 5.2. sICAM-1 Levels

Research has shown that soluble intercellular adhesion molecule-1 (sICAM-1), sE-selectin, and sP-selectin levels are influenced by ABO blood group. To determine whether the expression of these cell surface adhesion molecules is affected by ABO blood group, an analysis was conducted on 27 patients with hemophilia A. The study found no significant differences in the levels of sVCAM-1, sE-Selectin, or sP-selectin among different ABO blood groups (*p*  >  0.05). This result suggests that elevated sVCAM-1 levels in patients with hemophilia and hemophilic arthropathy are independent of blood group. However, caution is advised in interpreting these findings, as the patient sample may not be representative of the general population [33].

### 5.3. Other Soluble Biomarkers

Hemarthrosis results in cartilage and bone degradation, inflammation, and angiogenesis in patients with hemophilia A. Research has explored the relationship between various soluble biomarkers and these pathological symptoms. The biomarkers studied include C-terminal telopeptides of type I collagen, cartilage oligomeric matrix protein (COMP), tissue inhibitor of metalloproteinases-1 (TIMP-1), matrix metalloproteinases-3 and -9 (MMP-3 and MMP-9), vascular endothelial growth factor (VEGF), and chondroitin sulfate 846 epitope (CS846). Despite extensive investigations, no strong correlations between these biomarkers and MRI joint scores have been observed, except for a positive association between CS846 and MRI joint scores reported in the Oldenburg study [34].

Joint arthropathy in hemophilia A presents unique challenges because it is not a systemic disease, unlike rheumatoid arthritis (RA) and osteoarthritis (OA). This localized nature complicates the analysis of biomarkers, making it more intricate than in systemic joint diseases.

## 6. Bone Turnover Markers

Hemophilic arthropathy was originally characterized as a degenerative joint disease rather than an inflammatory one. An essential regulator of bone biology is the molecular triad consisting of osteoprotegerin (OPG), the receptor activator of nuclear factor κB (RANK), and RANK ligand (RANKL). This triad governs local bone turnover and is pivotal in triggering bone resorption induced by inflammation. RANKL, a transmembrane ligand, is primarily expressed on osteoblasts and stromal cells within the bone microenvironment. It is also produced by lymphocytes and synovial cells, contributing to osteoclastogenesis through a mechanism enhanced by several cytokines, such as TNF-α, IL-1, and IL-17, which collectively promote inflammation and bone resorption [6].

RANKL interacts with its receptor RANK, located on the surface of osteoclast precursors, leading to the differentiation and maturation of osteoclasts, which in turn fosters bone resorption. In this regulatory system, OPG acts as a decoy receptor for RANKL, competing with RANK for binding to RANKL. This competition effectively inhibits osteoclast differentiation, activity, and survival both in vivo and in vitro, thereby reducing bone resorption [35].

**Figure 1:**
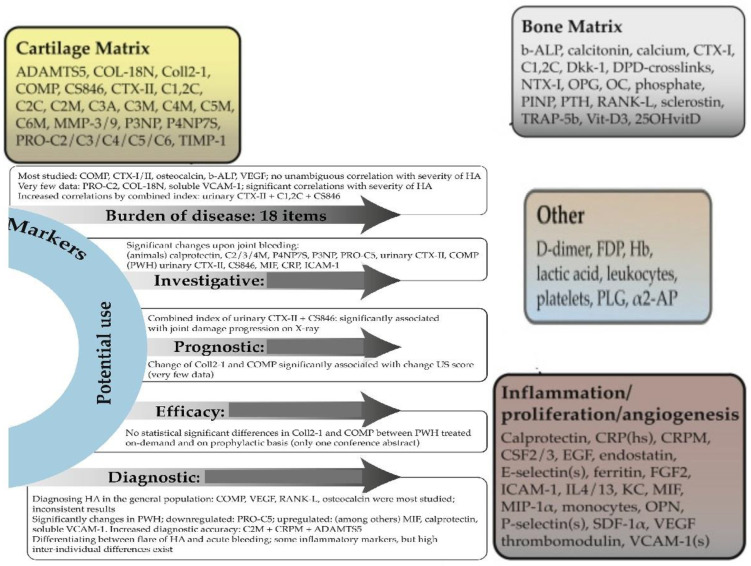
Biomarkers in HA [38].

In hemophiliacs, there is a notably high prevalence of osteoporosis, which is closely linked to the severity of arthropathy and is further exacerbated by HIV infection. This condition highlights a significant imbalance, where increased bone resorption is not adequately compensated for by bone formation. Research suggests that the OPG/RANK/RANKL triad could be a key regulator of bone remodeling in the synovial tissue of adult hemophiliacs. Decreased levels of OPG and strong expressions of RANK and RANKL have been observed, indicating a shift towards enhanced bone resorption (Figure 1).

The severity of hemophilic arthropathy has been shown to correlate strongly with instrumental findings such as the World Federation of Hemophilia (WFH) orthopedic joint scale, Petterson scores, and ultrasound (US) evaluations. Molecular markers of bone turnover in the synovial tissue of hemophiliacs clearly indicate osteoclastic activation, which is not counterbalanced by OPG. Strong expressions of RANK and RANKL are found in the synovium, regardless of the type of treatment, whereas the expression of OPG is significantly reduced in patients with hemophilic arthropathy. This almost complete lack of OPG expression implies that the balance of bone turnover is skewed towards osteoclastic activity and consequently, bone resorption [36,37].

Moreover, in vitro evidence suggests that reduced thrombin production leads to decreased thrombin-induced PAR-1 mediated proliferation of osteoblasts. These collective findings underscore the complexity of bone damage in hemophilic arthropathy, indicating that it is a multifactorial process. This complexity necessitates a comprehensive approach to understanding and treating bone damage in these patients, considering the intricate interplay of various molecular pathways involved in bone resorption and formation [39] (Table 3).

## 7. Future Perspectives

The shift from episodic treatment to prophylaxis marked a significant advancement in hemophilia care. The next goal should be to ensure long-term protection, as preventing arthropathy relies on effectively preventing bleeding—something that current treatments have not yet been able to consistently achieve. A key unmet need in the management and monitoring of hemophilic arthropathy is the absence of serum and synovial biomarkers to gauge disease activity. Identifying and validating such biomarkers would enhance decision-making. Research on synovial tissue in patients with recent-onset RA has revealed significant correlations between histological findings, transcriptomic profiles, and clinical responses to treatments. Similarly, synovial biomarkers derived from transcriptomic analysis could help identify predictors of therapeutic responses and potentially uncover new targets for personalized management of hemophilic arthropathy. The increased use of joint ultrasound (US) as a routine clinical examination in comprehensive care centers is anticipated to streamline workflows, enable prompt diagnosis of acute hemarthrosis, and enhance early treatment. Additionally, implementing point-of-care US in clinical practice and at the bedside should facilitate regular monitoring of arthropathy progression, provided that US techniques are standardized [38,40,41].

### 7.1. The Importance of Physical Activity in HA Pathogenesis

Another key role is linked to the degree of physical activity and machine-controlled motions. The health benefits of physical activity for the general population are also applicable to individuals with hemophilia. Maintaining adequate muscle tone helps prevent injuries and reduces the risk of joint bleeding. Physical exercise is particularly important for people with hemophilia as it enhances their quality of life. Numerous studies highlight the significance of physical training for improving health, aligning with recommendations from both the World Health Organization and the World Federation of Hemophilia. Specifically, video games used for rehabilitation (exergaming) have positively influenced patients’ attitudes towards exercise and have been shown to improve strength, coordination, and mobility. Additionally, motion capture (MoCap) sensors are increasingly utilized in medicine and physical therapy due to their availability and affordability compared to traditional 3D optical MoCap systems. The Kinect sensor, a popular MoCap option, has been successfully applied in various medical and rehabilitation contexts, including post-stroke limb rehabilitation, elderly exercise monitoring and fall prevention, range-of-motion evaluation in adhesive capsulitis, balance and postural control assessment and training, and virtual gyms for individuals with limited mobility [42].

### 7.2. The Importance of Animal Models in HA Pathogenesis

Factor VIII (FVIII) is among the most immunogenic biologics, and the development of inhibitors against FVIII poses a significant challenge to optimal care, leading to increased patient morbidity and mortality. Understanding the mechanisms that predispose individuals to or drive FVIII immune responses is crucial for clinical management. Early reports suggested that the α-CD20 monoclonal antibody rituximab could potentially eradicate FVIII inhibitors, but subsequent studies showing modest efficacy with rituximab monotherapy have tempered these expectations. This limited efficacy may be partly due to the persistence of rituximab-resistant FVIII^+^ B cell subsets and the potential exacerbation of autoimmune disease due to increased BAFF (B cell activating factor) levels following B cell depletion. Doshi et al. indicate that a single dose of α-mBAFF monoclonal antibody in naive hemophilia A (HA) mice prevents the development of inhibitors, even after immune reconstitution. In mice with pre-existing inhibitors, combining α-mBAFF with α-mCD20 significantly reduces or eliminates FVIII inhibitors and maintains long-term suppression of FVIII-specific plasma cells (PCs). Recent evidence points to marginal zone (MZ) B cells as key mediators of the initial FVIII immune response, which can quickly differentiate into short-lived antibody-secreting cells (ASCs). High-affinity antibodies generally arise from germinal center (GC) reactions, where B cell receptor (BCR) rearrangement leads to differentiation into PCs or memory B cells. Within GCs, T follicular helper (Tfh) cells release BAFF to support the selection of high-affinity GC B cell clones. The substantial reduction in inhibitor levels with preemptive α-mBAFF monoclonal antibody treatment is likely due to MZ B cells’ reliance on BAFF for survival and differentiation. We propose that these mice exhibit lower rates of GC B cell reactions and, consequently, fewer PCs and memory B cells, which promotes tolerance to FVIII. Importantly, these mice still mount a strong immune response to unrelated antigens, demonstrating the safety and specificity of this approach. These findings, along with data from enzyme replacement therapy studies, suggest that BAFF contributes to the immunogenicity of biotherapeutics. However, as some HA mice still developed high-titer anti-FVIII antibodies, further research is needed to determine if adjusting the dose or treatment duration could more effectively prevent inhibitor development [43].

Type II collagen degradation, indicated by C2M levels, may serve as a predictive marker for cartilage degradation and the development of arthropathy. In hemophilic rats, joint bleeding on day 0 did not initially affect serum C2M levels, but a significant increase was observed after a second bleed on day 14, which correlated with the severity of arthropathy on histology [44]. Other collagen markers, such as serum C4M and P4NP7S, significantly increased one day after the second joint bleed, while serum C3M levels significantly decreased. Serum PRO-C5 and P3NP increased significantly one week after both the first and second joint bleeds. In hemophilic mice, plasma C4M and PRO-C4 levels also significantly rose two weeks after induced hemarthrosis. In dogs with experimentally induced hemarthrosis, urinary CTX-II levels significantly increased from day two to seven (by 75% to 155%), and serum COMP levels rose by 46% from baseline to day two [45]. The idea that joint bleeds can trigger a systemic pro-inflammatory state and that inflammatory markers might be useful for detecting hemarthrosis was tested in hemophilic mice. It was found that serum calprotectin, a marker of residual inflammation, was significantly higher in mice with induced hemarthrosis at two and twelve weeks compared to control mice, where calprotectin was undetectable [46].

## 8. Plasminogen Activation and MMP-Mediated Joint Destruction as a Likely Mechanism for Joint Damage in HA

Although the existing literature supports the involvement of protease activation in the pathophysiology of HA, further investigation in experimental and clinical settings is needed. Nonetheless, several established characteristics of HA lend support to this hypothesis. Bleeding into the joint results in an influx of hemosiderin, which catalyzes the production of reactive oxygen species and triggers chondrocyte apoptosis. Type A synoviocytes absorb hemosiderin and release proinflammatory cytokines, such as IL-1α, IL-6, and TNF-α, creating a cytokine storm within the joint that exacerbates cartilage and bone damage. These cytokines also promote the recruitment and activation of monocytes and macrophages, which in turn stimulate the production of nitric oxide and proteases, including MMPs and uPA. An imbalance in hemostasis, characterized by low tissue factor (TF) and high thrombin-activatable fibrinolysis inhibitor (TAFI) levels, can lead to uncontrolled activity of uPA and tPA. This influx of blood exposes the synovium to high levels of plasminogen, setting the stage for uPA-mediated plasminogen activation and subsequent plasmin production. Fibrin deposition is frequently seen in the cavities of inflamed joints, and as a cofactor for tPA-mediated plasminogen activation, it facilitates substantial plasmin generation. Both uPA and plasmin can cleave pro-MMPs to produce active MMPs [44].

## 9. Diagnostic Markers Used in HA

Different studies compared biochemical markers between patients with HA and controls, focusing on serum COMP, VEGF, and bone markers like RANK-L and osteocalcin. Serum COMP findings were mixed: two studies reported higher levels in patients with HA, while one found lower levels. Cartilage and collagen markers also varied, with some (C2M, CTX-II, C4M) elevated and others (ADAMTS5, Coll2-1, C3A, C3M, C5M) decreased in hemophilia patients [47,48,49]. Most cartilage formation markers showed no difference between patients with HA and controls, except serum PRO-C5, which was lower in patients with hemophilia patients [50]. For bone metabolism, patients with HA had higher serum parathormone and lower 25-OH vitamin D in two studies. Serum osteocalcin and sclerostin levels showed conflicting results. Other markers, such as RANK-L, CTX-I, Dkk-1, and OPG, also had inconsistent findings [51,52]. Acharya et al. found a significant fourfold increase in plasma VEGF-A, SDF-1α, and MMP-9 levels in patients with hemophilia with joint disease. Other studies reported elevated levels of plasma MMP-9, SDF-1α, soluble VCAM-1, and several other markers in patients with hemophilia, though results for some markers were inconsistent. Three studies on acute joint bleeding in hemophilia found increased levels of serum D-dimer, ferritin, FDP, leukocytes, plasminogen, and VEGF, while plasma EGF, CSF2, IL4/13, FGF2, and MIP-1α were lower. Osteopontin was significantly higher in young patients with hemophilia with clinical synovitis [53,54].

## 10. Conclusions

In contrast to earlier views that depicted hemophilic arthropathy exclusively as a degenerative condition, recent and comprehensive research has uncovered a more intricate interplay of factors. These studies reveal that HA encompasses not only degenerative changes but also a significant inflammatory component, with synovitis emerging as a critical element in its pathophysiology.

This updated understanding of HA emphasizes that the condition is driven by a combination of joint degeneration and inflammatory processes rather than being solely degenerative. Synovitis, or inflammation of the synovial membrane, has been identified as a key player in the progression and severity of HA.

The recognition of these dual mechanisms opens up new avenues for therapeutic intervention. Targeting both the degenerative and inflammatory aspects of HA could lead to more effective treatment strategies.

Overall, this evolving perspective not only broadens the scope of potential therapeutic targets for HA but also underscores the importance of advanced diagnostic tools like imaging techniques in managing the condition more effectively. Currently, biomarkers hold scientific interest, but they offer no practical benefits to patients. As of now, they do not provide additional value. Our current understanding of biomarkers in hemophilia contributes to the scientific knowledge of pathophysiology, but it still has to prove its clinical relevance. Consequently, a multifaceted approach utilizing multiple biomarkers may provide a more effective and precise method for assessing the severity of joint arthropathy in patients with hemophilia A. By leveraging a panel of biomarkers, clinicians might better capture the complex and localized processes of joint damage and inflammation, leading to improved diagnostic and therapeutic strategies tailored to the specific needs of patients with hemophilia A.

## Figures and Tables

**Table 1:** This table summarizes the distinct aspects of HA.

Aspect	Hemophilic Arthropathy (HA)
**Basic Pathophysiology**	Chronic proliferative synovitis and cartilage destruction
**Synovial Characteristics**	Presence of metalloprotease enzymes, inflammatory cells, and cytokines (IL-1, TNF-α)
**Origin of Inflammation**	Triggered by blood-induced injury
**Role of Synovial Iron Deposition**	Primary factor initiating and sustaining inflammation and cell proliferation
**Impact on Synovial Macrophages**	Promotes inflammatory pathways of hemarthrosis and HA
**Unique Pathological Features**	Direct interaction of blood with synovial membrane and fibroblast-like synoviocytes (FLS)
**Challenges in Understanding Pathophysiology**	Incomplete understanding; complexities beyond iron deposition
**Consequence of Blood Presence in Joint**	Triggers inflammatory response; secretion of inflammatory mediators
**Long-term Effects on Joint Tissues**	Degeneration of articular cartilage, subchondral bone; synovial membrane hypertrophy
**Persistent Inflammatory State**	Interconnected with joint tissue degeneration

**Table 2:** Key cytokines and their roles in hemophilic arthropathy (HA).

Cytokine/Chemokine	Role in HA
TNFα	Induces catabolic processes in joints; regulates FVIIIa and TM expression (22)
IL-1β	Triggers NF-κB activation; enhances synovitis and cartilage degradation (4)
IL-6	Associated with macrophage polarization; implicated in inflammatory responses (11)
CX3CL1	Role in degenerative joint lesions; systemic elevation in patients with HA (4)
Progranulin (PGRN)	Protective against TNFα catabolic effects; potential biomarker (27)

**Table 3:** Role of biomarkers in HA.

Markers	Role in HA	Details
**Soluble Adhesion Molecules**	**sVCAM-1 Levels**	- sVCAM-1 levels do not differ significantly between patients with hemophilia A with or without hypertension. - Previous studies show elevated sVCAM-1 in patients with hemodialysis, especially with HCV. - sVCAM-1 levels increase significantly only during advanced fibrosis in patients infected with HCV, indicating potential as a marker for disease progression.
	**sICAM-1 Levels**	- ABO blood group does not significantly affect levels of sICAM-1, sVCAM-1, sE-selectin, and sP-selectin in patients with hemophilia A. - Elevated sVCAM-1 levels are independent of blood group.
	**Other Soluble Biomarkers**	- Various biomarkers studied (e.g., C-terminal telopeptides of type I collagen, COMP, TIMP-1, MMP-3, MMP-9, VEGF, and CS846). - No strong correlations with MRI joint scores, except a positive association between CS846 and MRI joint scores in the Oldenburg study. - Multifaceted biomarker approach recommended due to the localized nature of joint arthropathy in hemophilia A.
**Bone Turnover Markers**	**OPG/RANK/RANKL Triad**	- The OPG/RANK/RANKL system is crucial in bone remodeling and resorption. - RANKL promotes osteoclastogenesis, leading to bone resorption. - OPG acts as a decoy receptor, inhibiting osteoclast activity. - In patients with hemophilia A, reduced OPG levels and a strong RANK/RANKL expression shift the balance toward bone resorption, contributing to osteoporosis and arthropathy.
	**Bone Resorption in Hemophilia**	- Patients with hemophilia A have a high prevalence of osteoporosis, linked to arthropathy severity and exacerbated by HIV. - A strong correlation between hemophilic arthropathy severity and instrumental findings (WFH orthopedic joint scale, Petterson scores, and US evaluations). - Reduced thrombin production leads to decreased osteoblast proliferation, contributing to the complexity of bone damage in these patients.
**Cytokine/Chemokine**	**Role in HA**	
TNFα	Induces catabolic processes in joints; regulates FVIIIa and TM expression.	
IL-1β	Triggers NF-κB activation; enhances synovitis and cartilage degradation.	
IL-6	Associated with macrophage polarization; implicated in inflammatory responses.	
CX3CL1	Role in degenerative joint lesions; systemic elevation in patients with HA.	
Progranulin (PGRN)	Protective against TNFα catabolic effects; potential biomarker.	
